# Maternal health service utilisation of adolescent women in sub-Saharan Africa: a systematic scoping review

**DOI:** 10.1186/s12884-019-2501-6

**Published:** 2019-10-21

**Authors:** Tensae Mekonnen, Tinashe Dune, Janette Perz

**Affiliations:** 1 Translational Health Research Institute (THRI), School of Medicine, Western Sydney University, Penrith, NSW 2751 Australia; 2 School of Science and Health, Western Sydney University, Penrith, NSW 2751 Australia

**Keywords:** Adolescents, Teenage pregnancy, Maternal health care, Service utilisation, Sub-Saharan Africa, Antenatal care, Skilled birth delivery

## Abstract

**Introduction:**

Sub-Saharan Africa has the highest rate of adolescent pregnancy in the world. While pregnancy during adolescence poses higher risks for the mother and the baby, the utilisation of maternity care to mitigate the effects is low. This review aimed to synthesise evidence on adolescent mothers’ utilisation of maternity care in Sub-Saharan Africa and identify the key determinant factors that influence adolescent mothers’ engagement with maternity care.

**Method:**

A systematic review of scholarly literature involving seven databases: ProQuest, PubMed, EMBASE/Elsevier, SCOPUS, PsycINFO, CINAHL and Infomit was conducted. Studies published in English between 1990 and 2017 that examined Sub-Saharan adolescent mothers’ experiences of utilising biomedical maternity care during pregnancy, delivery and the post-partum period were included.

**Results:**

From 296 relevant articles 27 were identified that represent the experience of adolescent mothers’ maternal health service utilisation in Sub-Saharan Africa. The review indicates that maternal health service utilisation in the majority of Sub-Saharan African countries is still low. There is also a wide discrepancy in the use of maternity care services by adolescent mothers across countries in Sub-Saharan Africa.

**Conclusions:**

The review reveals that a significant number of adolescents in Sub-Saharan Africa do not access and use maternity services during pregnancy. Several factors from individual to systemic levels contributed to low access and utilisation. This implies that interventions targeting the women, their partners, healthcare professionals, communities and the organisations (local to national) are necessary to improve adolescent mother’s engagement with maternity care in Sub-Saharan Africa.

## Background

Adolescence is the transitional period from childhood to adulthood characterized by significant physiological, psychological and social changes [1]. Pregnancies that occur between the age of 10 and 19, in general, are referred to as an adolescent, or teenage pregnancy [2]. According to the World Health Organization (WHO), one in five adolescent girls worldwide give birth by the age of 18 and about 16 million girls aged between 15 to 19 years give birth every year. The figures rise to one in three girls in the poorest regions such as Sub-Saharan Africa and South East Asia [3]. These figures are alarming as the majority of adolescent pregnancies in Sub-Saharan Africa are unintended. Evidence suggests that several factors that range from individual to societal levels expose adolescent women to the risk of unwanted pregnancy [4].

Past research suggests that adolescent pregnancy and childbearing are associated with higher health risks to the mother and her baby [5]. For example, Solomon and Isehak (1999) reported higher rates of preterm deliveries, prolonged labour and cephalic pelvic disproportion among teenage girls compared to older women [6]. Other researchers also showed that the risk of death during pregnancy and childbirth is higher among teenage mothers [2, 7]. In addition to the mother, adolescent pregnancy also poses a risk to the baby. Evidence suggests that babies born from adolescent mothers are more likely to have low birth weight [7] and Apgar score [8] and to be admitted to an intensive care unit [9]. Babies born in low - income countries from adolescent mothers also face 50% risk of stillbirth or dying before they are 1 month old in comparison to mothers whose age is above 20 [3]. These findings suggest that while reducing the rate of teenage pregnancies is ideal a concurrent goal is to support teenage mothers towards a safe and healthy pregnancy, delivery and baby through increased access and utilisation of skilled maternal health care [10].

The utilisation of mainstream maternal health services has a significant impact on the reduction of death and morbidity through early detection of danger signs and management of potential complications [11]. Maternal health care encompasses a range of services including antenatal care, skilled birth delivery care, and postnatal care. The provision of these services by formally trained and accredited health care professionals improves pregnancy outcomes significantly [10, 12]. Research has demonstrated that when compared to women who did not receive skilled maternal health care those who did experience fewer complications and discomforts during pregnancy, underwent less invasive biomedical intervention during the birth and adjusted better to postnatal life and childrearing [13, 14].

Despite having these benefits, the utilisation of maternal health services by adolescent women is generally poor. For example, Magadi states that adolescent mothers in Sub-Saharan Africa receive inadequate ANC and have non-professional deliveries [15]. Another study suggests that only a quarter of adolescent women in Nigeria received safe delivery care [11, 15, 16]. The use of postnatal care (PNC) is also poor with only a third of adolescent mothers receiving the service in Sub-Saharan Africa [16]. Although there is a wide range of factors that influence maternal health care utilisation amongst teenage mothers in sub-Saharan Africa the evidence base is minimal. However, it is well established and reinforced by the Sustainable Development Goals that adverse outcomes for mothers, children, communities and countries are inevitable when women cannot utilise maternal health care services [17]. Even so, the majority of research in this area fails to engage theoretical frameworks or holistic models towards the development of strategies to encourage a systemic approach to the problem. In order to contribute to holistic recommendations, this systematic review uses the socioecological model to help improve maternal health care utilisation amongst teenage mothers in Sub-Saharan Africa.

Currently, there exists a number of systematic reviews that examine the utilisation of maternal health services by adolescents. Some of these have focused on only ANC [18] and others considered all women in developing counties [19] or synthesised country - specific evidence on the topic [20]. In Sub-Saharan Africa, although extensive research has been carried out on adolescent mothers’ utilisation of maternity care during pregnancy, delivery and postnatal periods [15], no single study synthesised them to explore all aspects of adolescent mothers’ healthcare-seeking behaviour. This systematic review, therefore, aims to fill this gap and make recommendations to policymakers, programme planners and clinicians, to improve the maternal health of adolescent women in Sub-Saharan Africa. This is significant to achieve the United Nation’s (UN) target to reduce maternal mortality to less than 70 per 100,000 births under the Sustainable Development Goal 3 by 2030.

## Methods

A rigorous systematic review was undertaken to locate studies that examined antenatal care, skilled birth delivery and PNC service utilisation of adolescent mothers in Sub-Saharan Africa.

### Search strategy

Seven electronic databases were searched to identify potential articles that align with the review objective: ProQuest, PubMed, EMBASE/Elsevier, SCOPUS, PsycINFO, CINAHL and Infomit. Table 1 below presents the details of the search strategy including keyword combinations. A step-by-step search strategy was employed in this review. First, the first author identified relevant keywords by undertaking an initial MEDLINE search. The list and combinations of keywords were discussed, modified, and approved by the second author. A second search was then conducted across the identified databases and the results were downloaded and saved in an Endnote library for further screening and examination. Finally, studies conducted in Sub-Saharan Africa; involving quantitative, qualitative or mixed methods designs; focused on adolescents’ utilisation of maternal health care; and written in English were included.

**Table 1:** Summary of the inclusion/exclusion criteria and keywords

Location	Sub-Saharan Africa	Out of Africa	Sub-Saharan Africa (Document text)
Language	Written in English	Other languages	Select for English only
Time	Any	None	N/A
Population	Literature which include teenagers	Literature which doesn’t focus on teenagers	Teenagers (Abstract) or adolescent (Abstract) or young age (Abstract) or youth (Abstract)
Phenomena/Target	Studies concerned with maternal health care utilisation by teenagers which includes; ANC, Skilled birth delivery and postnatal care	Not concerned with maternal health care utilisation by teenagers which includes ANC, skilled birth delivery and postnatal care	AND Antenatal (Title) OR ANC (Title) OR Matern* (Title) OR Skilled birth delivery (Title) OR Institutional delivery (Title) OR Skilled birth OR PNC (Title) OR Pregnancy care (Title) OR Prenatal care (Title) OR Pregnancy care
Study/literature type	Published primary research including qualitative, quantitative and mixed method designs	Published literature which don’t include qualitative, quantitative and mixed methods of data collection and analysis	N/A

### Data synthesis and reporting

A standard extraction template was used to capture data relevant to the review objective. Prevalence rates for ANC, skilled birth delivery and PNC were extracted from each study and grouped and analysed. Comparisons across countries and studies were then made. Factors that influence the use of maternal health care services were polled and analysed together through a socio-ecological lens to have a holistic understanding of the issue. Finally, a narrative synthesis and interpretation of the data was undertaken.

### Quality assessment

The quality of the included studies that used quantitative data was performed using the *International Society for Pharmacoeconomics and Outcomes Research (ISPOR) Good Research Practices for Retrospective Database Analysis Checklist*. This tool has also been used in previous similar studies. The included studies were assessed across 17 criteria on a three-level scale. Items were awarded 0 if the “criterion was not met”, 1 if the “criterion was partially met” and 2 “criterion was fully met.” The maximum score was set at 34 (100%). Studies with a cumulative score of 70% or more were classified as high quality, medium quality if the study scored from 50 to 70%, and low quality if the study scored less than 50%.

### Theoretical framework

As noted, socioecological theory provides a useful framework for understanding the effects of multiple levels of influence (e.g., individual, family, peer/community and social system) on maternal health service utilisation [21]. According to the theory, use of maternal health care by an adolescent is influenced by a range of factors, such as individual experiences and attitudes, to the system level which is mainly related to policies and organizational level factors [22]. By providing a comprehensive view, socioecological theory helps program planners and providers identify the main social determinant factors and strategies to mitigate health problems [23].

## Results

### Search results

**Fig. 1:**
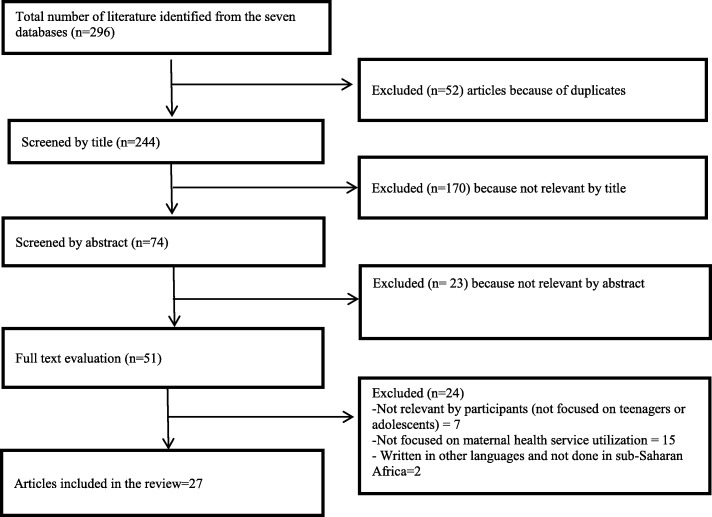
Article Selection Process

Of the 296 potentially relevant articles identified through a database search, 27 were included in this systematic review (see Fig. 1).

### Characteristics of included studies

As demonstrated in Table 2, five studies were conducted in Kenya, three in South Africa, and Zimbabwe, Nigeria and Malawi each having two studies. Most studies (85%) focused on the ANC experiences of women with 13 and 7 studies concentrated on skilled birth delivery and PNC services respectively. Twenty-one studies used quantitative methods, four used qualitative approaches and the remaining (*n* = 2) involved mixed-methods design. Most quantitative studies used bivariate and multivariate logistics regression analysis while qualitative studies involved thematic analysis. Three articles used theoretical approaches including the health belief model, Guba’s model of trustworthiness, phenomenology and Donabedian’s model. Most of the quantitative studies (*n* = 14) used data from the demographic and health surveys (DHS), which is retrospective, 8 of the reviews used primary data sources, and three were follow-up studies. Of the studies that used retrospective data one study used 3 years of data to minimize recall bias.

**Table 2:** Characteristics of included studies

No	Authors/Year	Country of study	Sample size	Type of care	Outcome measured	Study design/data collection method	Data Analysis	Theoretical Approach
1	[24]	Ethiopia	994 women aged between 14 and 19	ANC	Factors influencing the utilisation of ANC	Survey/ Quantitative	Bivariate and multivariate analyses	N/A
2	[25]	Kenya	1675 women aged 15–24	ANC	Relationship between timing of first (ANC) visit and type of delivery assistance	Survey/ Quantitative	Multivariate logistic regression	N/A
3	[16]	Nigeria	2434 married adolescents aged 15–19	ANC, Delivery and PNC	Factors associated with maternity services	Survey/ Quantitative	Pearson chi-square test and binary logistic regression	N/A
4	[26]	Mali	1646 adolescent mothers age 15–19	ANC, Delivery and PNC	Factors associated with the utilisation of maternal services	Survey/Quantitative	Bivariate and multivariate analyses	N/A
5	[27]	Zimbabwe	80 adolescent mothers aged 19 or younger	ANC	Factors influencing adolescents‘ non-utilisation of ANC services	Quantitative	Descriptive (Frequency and proportion	Health belief model
6	[28]	Niger	934 adolescent mothers aged 15–19	ANC, Safe delivery and Immunization	Potential factors associated with the utilisation of MCH care services	Survey/Quantitative	Multivariate logistic regression models	N/A
7	[29]	Malawi	615 adolescents aged 10–19 years	ANC	Why antenatal care (ANC) programs for adolescents may need to be improved?	Quantitative	Chi-square test and binary logistic regression	N/A
8	[30]	Tanzania	*N* = 440	ANC	Factors influencing early and late ANC attendance	Quantitative	logistic regression models	N/A
9	[31]	Sub-Saharan Africa	Data from 20 countries (total of 74,559 births)	Safe delivery	Perceived size of newborn and caesarean section deliveries among teenagers	Quantitative and comparative study	Multiple logistic regression models	N/A
10	[32]	Swaziland	*N* = 33 pregnant adolescent mothers	Safe delivery and PNC	Quality of maternity care	Quantitative and qualitative	Content analysis	Donabedian’s (1988) model
11	[33]	South Africa	*N* = 20 aged 12—19 years	ANC	Communication in ANC	Qualitative	Tesch‘s method of data analysis (Creswell, 2008:186)	N/A
12	[34]	Zimbabwe	*N* = 40	ANC and PMTCT	Gaps in service uptake	Quantitative and retrospective analysis	Multivariable binomial regression analysis	N/A
13	[35]	Nigeria	*N* = 114	ANC, safe delivery and PNC	Prevalence of pregnancy Complications and interventions	Quantitative	Fisher’s exact test	N/A
14	[36]	Kenya	898 female adolescents between 15 and 19 years	ANC, skilled birth attendance and PNC	Factors that impact maternity care utilisation	Survey/ Quantitative	Bivariate and multivariate analyses	N/A
15	[37]	Sudan	459	ANC and safe delivery	Risk of anaemia, operative delivery, and perinatal complications	Quantitative	Chi-square test and Fisher’s exact test	N/A
16	[38]	Kenya	278 adolescent mothers aged 19 and younger	ANC	Factors affecting utilisation of ANC	Survey/ Quantitative	Bivariate and multivariate analyses	N/A
17	[39]	South Africa	383	ANC	Factors that influence ANC uptake	Quantitative and qualitative	Bivariate and multivariate analyses	N/A
18	[40]	Malawi	2160 adolescent married women aged 15–19	ANC and PNC	Factors that influence ANC and PNC	Survey/ Quantitative	Bivariate and multivariate analyses	N/A
19	[15]	Sub-Saharan Africa	Data from 21 DHS countries were pooled	ANC and Delivery	Association between maternal age ANC and delivery care	Survey/Quantitative	Bivariate and multivariate analyses	N/A
20	[41]	Tanzania	Adolescents aged 15–20 years	ANC and RH service	Barriers to SRH care	Qualitative/focus group	Thematic analysis	Phenomenology
21	[42]	Kenya	393 adolescents aged 15–19	ANC, skilled birth attendance and PNC	Factors that influence ANC and PNC.	Quantitative	Multilevel logit models	N/A
22	[43]	Uganda	*N* = 18 key informants (health workers and community leaders)	ANC	Factors affecting utilisation of teenager friendly ANC	Qualitative	Thematic analysis	N/A
23	[11]	Developing countries		ANC, safe delivery and immunisation.	Utilisation of ANC, delivery and PNC	Survey/Quantitative	Bivariate and multivariate logistic regression	N/A
24	[44]	Kenya	301 adolescent mothers aged 15–19 years	ANC, safe delivery and PNC	Maternity care utilisation	Survey/Quantitative	Bivariate and multivariate analyses	N/A
25	[45]	Central and west Africa	Adults aged 20–49 and Adolescent aged 10–19	ANC	HIV testing in ANC	Survey/Quantitative	Bivariate and multivariate analyses	N/A
26	[46]	South Africa	18 adolescent,15–19 yrs	ANC and PNC	Maternity care perception	Qualitative		Grounded theory
27	[47]	Kenya	13–19 years	ANC	Factors influencing utilisation of antenatal care service among teenagers	Survey/Quantitative	chi square test and logistic regression	N/A

### Study quality

As shown in Table 3, the quality assessment tool evaluated the included studies based on the following four major components: a) objectives, b) methods, c) results, and d) discussion. A total of 17 specific quality assessment sub-criteria where weighted according to a modified version of the *International Society for Pharmacoeconomics and Outcomes Research (ISPOR) Good Research Practices for Retrospective Database*. The checklist sub-criteria provided an overall indication of a study’s quality level as strong (score = 2), moderate (score = 1) and weak (score = 0). Study quality varied significantly across the studies. Overall, 11 studies were identified as high quality (score ≥ 70%), eight were of moderate quality (score between 50 and 69%), and the remaining three papers were poor in quality (score < 50%).

**Table 3:** Quality assessment for included studies

Component	Item	Quality criteria description	Banke-Thomas, 2016	Magadi, 2007	Brabin, 1998	Magadi, 2002	Reynolds, 2006	Ebeigbe, 2007	Magadi*,* 2007	Elhassan*, 2009*	Chaibva, l,2009	Alemayhue, 2010	Birungi, 2011
Objectives	1	State specific objectives, including any pre-specified hypotheses	2	2	2	2	2	1	2	2	2	2	2
Methods													
Study design	2	Present key elements of study design early in the paper	2	2	1	1	2	1	2	1	2	1	1
Setting	3	Describe the setting, locations, and relevant dates, including periods of recruitment, exposure, follow-up, and data collection	2	1	2	2	2	2	1	2	2	2	2
Participants	4	Give the eligibility criteria, and the sources and methods of selection of participants	2	1	1	2	2	1	2	1	2	2	2
Variables	5	Clearly define all outcomes, exposures, predictors, potential confounders, and effect modifiers.	2	2	2		1	1	2	1		1	1
Data sources/ measurement	6	For each variable of interest, give sources of data and details of methods of assessment (measurement).	2	2	1	1	2		2		2	1	1
Bias	7	Describe any efforts to address potential sources of bias	2	1	1		1		1		1	1	1
Study size	8	Explain how the study size was arrived at	1		1	2	2			1	1	1	1
Quantitative variables	9	Explain how quantitative variables were handled in the analyses. If applicable, describe which groupings were chosen and why	2	2	2	1	2	1	2	1	2	2	2
Statistical methods	10	Describe all statistical methods, including those used to control for confounding	2	2	2		2	1	2	1	1	1	1
	11	Explain how missing data were addressed	1	1	1	1	1	1	1	1			1
Results													
Participants	12	Report numbers of participants and study and response rate	2	1	1		2	1	1	1	2	1	1
Descriptive data	13	Give characteristics of study participants (eg demographic, clinical, social) and information on exposures and potential confounders	2	1	2	1	2	2	2	2	1	2	2
Main results	14	Give unadjusted estimates and, if applicable, confounder-adjusted estimates and their precision (eg, 95% confidence interval). Make clear which confounders were adjusted for and why they were included	2	2	1		2	1	2		1	1	1
Discussion													
Key Results	15	Summarise key results with reference to study objectives	2	2	2		2	2	2	1	1	2	1
Limitations	16	Discuss limitations of the study, taking into account sources of potential bias or imprecision. Discuss both direction and magnitude of any potential bias	2	1	1		2	1	1		2	1	2
Interpretation	17	Give a cautious overall interpretation of results considering objectives, limitations, multiplicity of analyses, results from similar studies, and other relevant evidence	2	1	1	1	2	1	2	2	1	2	2
		Quality score	32	26	24	16	31	17	27	17	23	23	24
		% Quality score	94	77	70	47	91	50	79	50	67	67	70
Component	item	Quality criteria description	Ochako, 2011	Gross, 2012	Rai, 2012	Rai l, 2013	Singh, 2006	Rai, 2014	Worku, 2016	Banke-Thomas.2017	Helleringer, 2017	Musarandega, 2017	Ronen, 2017
Objectives	1	State specific objectives, including any pre-specified hypotheses	2	2	2	2	2	2	2	2	2	1	1
Methods													
Study design	2	Present key elements of study design early in the paper	2	1	2	1	1	1	1	1	2	2	2
Setting	3	Describe the setting, locations, and relevant dates, including periods of recruitment, exposure, follow-up, and data collection	2	2	2	2	1	1	2	2	1	1	2
Participants	4	Give the eligibility criteria, and the sources and methods of selection of participants	1	2	2	1	2	1	2	2	2	2	2
Variables	5	Clearly define all outcomes, exposures, predictors, potential confounders, and effect modifiers.	1	1	2	2	2	2	1	2	1	2	1
Data sources/ measurement	6	For each variable of interest, give sources of data and details of methods of assessment (measurement).	2	1	1	2	1	2	1	2	1	2	1
Bias	7	Describe any efforts to address potential sources of bias	1		1	1	1	1	2	2	1		1
Study size	8	Explain how the study size was arrived at	2	1	2	2	1	2	1	1	2	2	1
Quantitative variables	9	Explain how quantitative variables were handled in the analyses. If applicable, describe which groupings were chosen and why	1		2	1	2	2	1	2	1	2	1
Statistical methods	10	Describe all statistical methods, including those used to control for confounding	1	1	1	1	1	2	1	2	1	1	1
	11	Explain how missing data were addressed	1		1		1						
Results													
Participants	12	Report numbers of participants and study and response rate	2	2	1	1	2	1	1	1	1	1	1
Descriptive data	13	Give characteristics of study participants (eg demographic, clinical, social) and information on exposures and potential confounders	2	1	2	2	2	2	2	2	1	2	1
Main results	14	Give unadjusted estimates and, if applicable, confounder-adjusted estimates and their precision (eg, 95% confidence interval). Make clear which confounders were adjusted for and why they were included	1		1	1	1	1	1	2	1	1	1
Discussion													
Key results	15	Summarise key results with reference to study objectives	2	2	2	2	2	1	2	2	2	2	1
Limitations	16	Discuss limitations of the study, taking into account sources of potential bias or imprecision. Discuss both direction and magnitude of any potential bias	1	1		1	1	1	2	1	1	1	1
Interpretation	17	Give a cautious overall interpretation of results considering objectives, limitations, multiplicity of analyses, results from similar studies, and other relevant evidence	1	2	2	2	1	2	2	2	1	1	1
		**Quality score**	25	19	26	24	24	24	27	29	21	23	19
		% Quality score	73	55	76	70	70	70	79	85	61	67	55

### Major findings

Following the analysis of the extracted data from individual studies the synthesised results regarding the utilisation and socioecological factors related to maternal health care services are presented in four categories: *Antenatal Care Utilisation*, *Skilled Birth Delivery Utilisation*, *Postnatal Care Utilisation* and *Factors Influencing Maternal Health Care Utilisation*.

### ANC utilisation

**Fig. 2:**
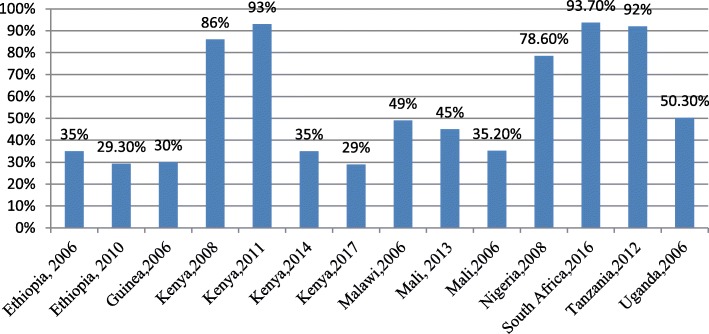
ANC attendance during pregnancy by adolescent mothers in Sub Saharan Africa (ANC1+)

**Fig. 3:**
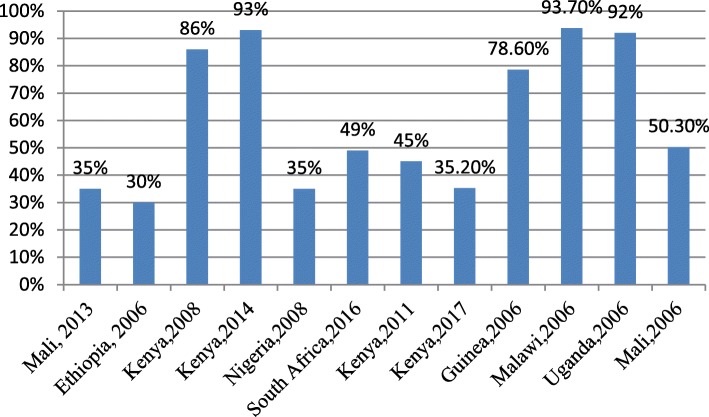
Four or more ANC visits during pregnancy by adolescent mothers in Sub-Saharan Africa

Twenty-two out of 27 studies reported on adolescent women’s engagement with ANC services. Figures 2 and 3 below summarise the proportion of adolescent women who had accessed ANC at least once and those who accessed ANC four or more times during pregnancy. This proportion ranges from 29% in Ethiopia to 93% in South Africa and Kenya for at least one ANC visit, and from 30% in Ethiopia to 93.7% in Malawi 4+ visits.

Five studies also reported on the timing of ANC visits with the majority saying late attendance to ANC (late second trimester and after that) care by adolescent women [15, 25, 30, 35]. However, Musarandega and colleagues said that adolescent women were more likely to present for ANC in the first trimester compared to older women [34]. Being a first-time adolescent mother was also said to have a positive association with first trimester ANC visits [30].

### Skilled birth delivery utilisation

**Fig. 4:**
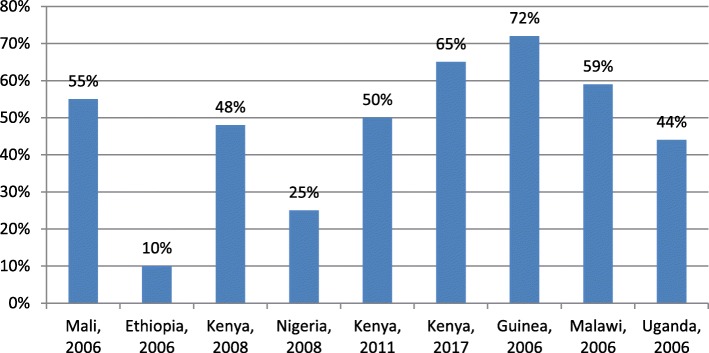
Proportion of adolescent women who accessed skilled care during delivery in Sub-Saharan Africa

Thirteen out of twenty-seven studies described the use of skilled birth delivery services by adolescent mothers during the birth of their babies. Figure 4 below shows the proportion of adolescent mothers who received skilled care during delivery. This proportion ranges from 10% in Ethiopia to 72% in Guinea.

### PNC utilisation

**Fig. 5:**
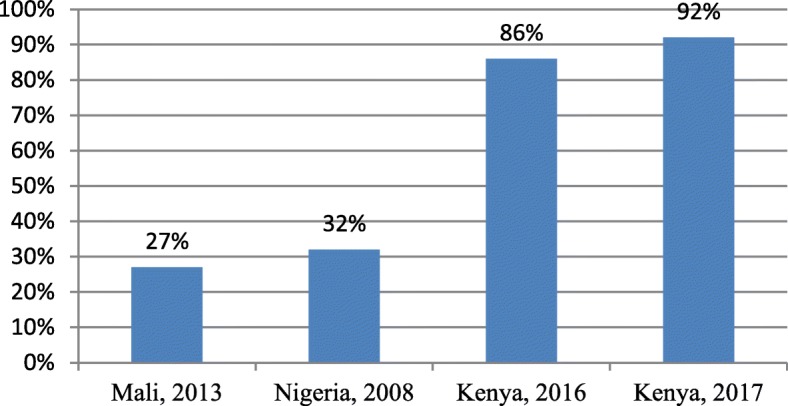
Postnatal care utilisation by adolescent mothers in Sub-Saharan Africa

Only seven studies out of 27 described PNC. One study reported that adolescent mothers were more likely to have babies with low Apgar scores and low birthweight compared to older mothers [37]. Finally, one study indicated that adolescents do not receive optimal maternity care, and are not followed up adequately immediately after delivery [32]. Figure 5 below indicates the use of PNC service by adolescent mothers in sub-Saharan Africa.

### Factors affecting maternal health care utilisation

**Fig. 6:**
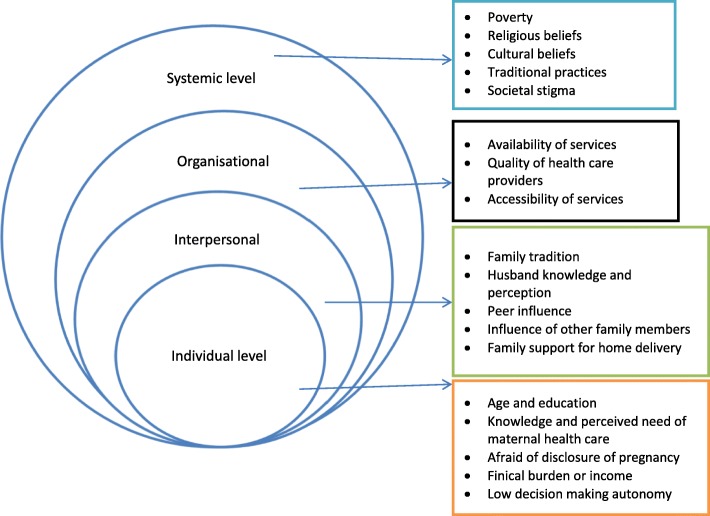
A socioecological analysis of factors influencing adolescent mothers’ engagement with maternity care in Sub-Saharan Africa

Twenty-two out of 27 studies reported on the factors affecting adolescent women’s utilisation of maternal health care services in Sub-Saharan Africa. Figure 6 below provides a socioecological summary of these factors across four levels.

### Individual level

At the individual level, factors such as educational status, age, residence, economic status and knowledge and perceived need of maternal health care were identified to influence the utilisation of maternity care by adolescent mothers in Sub-Saharan Africa. The majority of studies included in this review reported that women’s education is the most significant predictor for the utilisation of maternal health services with women having a secondary and above level of education more likely to seek maternity care than those who had no education [16, 25, 28, 29, 36, 40, 44]. Alemayehu and colleagues explain that education helps women develop greater confidence to make decisions regarding their health [24]. Furthermore, seven studies reported that adolescent women who were residing in urban areas were more likely to access maternal health care than those living in rural areas [25, 36, 40, 48]. Some studies further explained the urban-rural discrepancy. According to the reviewed studies the reason for rural–urban disparity in utilisation of health services can be explained by variations in access to maternal health resources, where urban location often tend to be more advantaged relative to rural areas [36] and rural women influenced by attitude, belief and social norms that discourage to use maternal health care [24–26, 42].

Furthermore, some studies identified that although adolescent women knew the benefits of having ANC and delivering with the assistance of a skilled provider, they did not attend ANC care and received skilled birth delivery due to fear of disclosing pregnancy and lack of money for ANC registration [27]. Similarly, adolescent women who have had more than three children with less than 2 years between pregnancies were less likely to utilize postnatal services compared to adolescent women with fewer children and or more spacing [40]. Other factors that were found to be positively associated to the use of maternal health service among adolescent mothers include mass media exposure [36, 40, 44], low parity [25, 38] and employment status [38].

### Interpersonal or family level factors

The review also identified interpersonal level factors that influence adolescent women’s utilisation of maternal health care which includes family traditions, husband’s knowledge, education and perceptions, peer influence and the influence of other family members. Some studies that captured health provider perspectives reported that adolescent mothers often experienced unintended pregnancy which further complicated their maternal health - seeking behaviour and journeys. In such situations, teenage mothers frequently experienced rejection from their partner and social isolation [41]. Following these outcomes, the more fortunate teenage mothers were allowed to stay with their parents albeit with limited emotional, financial and social support [30]. Other young mothers may not be disowned, asked to leave and/or forcibly removed from their home and community [41]. These difficulties are the result of negative sociocultural perceptions of unplanned teenage pregnancy often reinforced by religious, traditional and or spiritual beliefs held within families and communities [41]. Also, Rai and Singh suggest that the chances of accessing maternity care were lower among those teenage mothers who reported the birth as unwanted [40]. These factors imply that teenage mothers may not able to access and utilise available reproductive health care services at any point during their pregnancy [43]. However, those adolescent mothers who were married [25] and had educated husbands [40] were significantly likely to use maternal health services.

Adolescent women’s interaction with health providers had both positive and negative effects on their experience of accessing maternity care. In some studies, health providers were described to be “harsh,” “judgmental” and “not trustworthy” with regards to confidentiality. This discouraged adolescent women from accessing maternity care [41, 49]. Health providers also reported a lack of training and supervision related to adolescent sexual and reproductive health services [50] which made it challenging to provide adolescent - friendly reproductive health services. In some instances, HCPs were reluctant to provide reproductive health care due to lack of clarity in legislation and fear of condoning adolescent sexual activity [49].

### Organizational level

The review also identifies several organisational level factors that positively or negatively influence adolescent mothers’ utilisation of maternity care in Sub-Saharan Africa. Although HCPs view teenage mothers as a special group that requires great attention, the lack of teenage friendly services constrained their utilisation of ANC care, with adolescent women attending adult oriented ANC services that do not provide adequate privacy, time or specific training in teenage pregnancy to HCPs [41, 43]. Due to a lack of trust for health providers, pregnant teenage mothers were also reported to experience communication barriers with health providers when accessing ANC care [33]. Others reported a severe shortage of HCPs leading to long waiting times for pregnant adolescent women in ANC services and poor satisfaction with the service [39, 43, 46]. Distance from the health facility [39] was also reported to constrain adolescent mothers engagement with maternity care. Although several adolescent mothers represented in the studies felt the need for information to prepare themselves for pregnancy and motherhood responsibilities better; many did not receive this education due to the lack of services and resources [46]. HCPs also corroborated the adolescent women’s claims that they had a shortage of guidelines and educational materials to provide maternal health education to adolescent mothers [50].

Four additional studies identified an association between ANC and skilled birth delivery care. While Ochako and colleagues [25] reported the positive impact of first trimester ANC visits on skilled birth delivery utilisation other studies [16, 28, 40, 42] identified the influence of frequent ANC visits were attending at least four times significantly influenced young women’s utilisation of skilled birth delivery care. Conversely, one study reported that adolescent mothers in rural Malawi who attended ANC during the first, and early in the second, trimester were less likely to attend skilled birth delivery [29]. Others revealed that having at least four antenatal care (ANC) visits [42] increased utilisation of safe or skilled birth delivery care [40] and had shown a substantial effect on the use of PNC service. These findings suggest the need to integrate maternal health care service to better improve adolescent mother’s utilisation of maternity care [28]. This is also important for improving child health as children whose mothers received maternity care were more likely to receive full immunization [28].

### Systemic level

The included studies identified few systemic level factors that influence the utilisation of maternal health services by adolescents. For instance, the utilisation of all three maternal healthcare services was observed to increase with the increase in wealth quintile [16, 24, 25, 36, 44] where adolescent women in the middle and high wealth categories were significantly likely to use maternal health care services [16, 25, 28, 36, 44]. One study reported on the barriers to accessing ANC care by adolescent mothers which include ANC with traditional birth attendants [27] that reduced the perceived need for adolescent women to access skilled maternity care. Unmarried pregnant adolescents felt ashamed and experienced stigma in front of their peers, neighbours and even relatives and as a result was afraid to visit health facilities [41]. In addition, Islamic adolescents appeared to have a higher probability of using maternal health services compared to Catholic adolescents [40]. These findings suggest that multiple factors influence adolescent mothers’ utilisation of maternal health care, and multiple and multilevel interventions that address all levels of the socioecological model are needed to improve adolescent mothers’ health and wellbeing in Sub-Saharan Africa.

## Discussion

Our review indicates that maternal health service utilisation in the majority of Sub-Saharan African countries is still low. The review also suggests that there is a wide discrepancy in the use of maternity care services by adolescent mothers across countries in Sub-Saharan Africa. A socioecological analysis of the data also identified several factors at individual, interpersonal, organisational and community levels that influence the use of maternity care, suggesting the need for multiple and multilevel interventions targeting this group. This is critical given that young women are at higher risk of experiencing complications during pregnancy, childbirth and the postpartum period which may lead to maternal and child mortality or a lifetime of poor health outcomes.

Although the WHO classifies adolescent pregnancy as high risk and recommends close monitoring by skilled professionals from the beginning of the pregnancy [3] most of the studies reported that adolescent girls did not achieve the minimum four ANC visits during pregnancy and instead initiated ANC visits late in the second or third trimesters. This is attributed to inadequate knowledge on essential aspects of ANC service and misconceptions (feeling well and baby is kicking). However, one study done in Kenya reported that teenage women had good general knowledge of the common danger signs during pregnancy [48]. This can be due to improved awareness of ANC services in that specific country as shown by the high percentage of women reporting at least one ANC visit.

The socioecological analysis indicated that the majority of the studies reported that women’s and their partner’s education is the main factor influencing the use of ANC, delivery by skilled professionals and PNC. This is in line with the findings of previous studies in Sub-Saharan Africa and elsewhere [20]. Past research suggested that educated men and women are more likely to have knowledge about the benefits of skilled maternity care and the required empowerment to access care [20]. Effort is needed to strengthen schooling by expanding non-formal educational options for adolescent girls and encouraging adolescent girls to come back to school after they give birth. This is significant given the difficulty of re-entry of adolescent mothers into school due to traditional and institutional ideologies in Sub-Saharan African countries [51]. Clearly, effort is needed from governmental and non-governmental organizations to change such barriers as they perpetuate cycles of disadvantage by restricting young women’s access to education, future employment, financial stability and therefore empowerment – key factors that increase maternal health care access and utilisation [52]. Further, covering direct extraneous costs such as books, school uniforms, and transportation and indirect (opportunity) costs of schooling were also other ways to increase school attainment among teen mothers [53].

Economic status of the adolescent mother was also found to be positively associated with the use of maternal health care with adolescents in the wealthier group reporting better engagement with maternity care compared to the poorer ones. Muldoon and colleagues suggested that poor adolescents lack resources to spend on healthcare. Also, poor adolescents are more likely to be disengaged from social networks, thus, less likely to be reached by programs aiming to improve maternal health service utilisation of adolescent mothers [54]. Therefore, poverty reduction interventions for adolescent girls may have a more transformative power and longer-lasting impacts on their future sexual and reproductive health outcomes.

Social and cultural beliefs, and practices regarding pregnancy and childbirth have a significant influence on maternal health [55]. For example, in Sub-Saharan Africa and South Asia, religion often influences beliefs, norms and values related to the use of skilled maternal health services [56]. For instance, some women and societies may believe that birth is a test of endurance, care-seeking is a sign of weakness or that health facility delivery is only for prolonged and complicated labour [56, 57]. In addition, teenage women may be less familiar with biomedical maternity services and more exposed to traditional birth attendants especially in rural and remote areas of Sub-Saharan Africa [58] Wakefield and colleagues have also suggested the use of media to target social norms and beliefs at a community level [59]. For example, television, radio, print media can be used to disseminate consistent messages promoting the use of maternal health services and increase discussion of these issues within the community. However, previous studies have reported that many Nigerian women, particularly those in rural areas, rate the services of the traditional birth attendants (TBAs) as being of higher quality than that of medical healthcare practitioners, particularly with regards to interpersonal communications and relationships [60]. TBAs have been reported to be more considerate and to provide more compassionate care [61]. Improving health providers’ competency including their ability to communicate to adolescent clients is therefore essential to increase adolescent mothers’ utilisation of skilled maternity care [61].

Special efforts are needed to focus on creating awareness on the benefit of maternal health care when adolescents attend ANC. In order to do this strengthening staffing is a priority, since shortage of staff is a significant barrier to adequate service delivery [41]. Furthermore, increasing access to family planning for adolescent girls to prevent early childbearing and poor maternal health outcomes is required. Importantly, the provision of family planning education and services should be part of an integrated system between schools, communities, health organisations, and governments to ensure that young women are empowered to make decisions about their sexual and reproductive health as soon as possible instead of during the post-natal period when such counselling is provided. Also, relevant efforts are needed to involve community leaders and other key stakeholders as agents of change [62]. Such organizational change is especially needed in rural areas given the many barriers that restrict these women from accessing and therefore utilizing maternal health care [63, 64].

Given that adolescents girls are not always comfortable receiving ANC service together with older women, because they fear judgments and ridicule from them, establishing separate maternal care services for adolescent girls can increase ANC utilisation. Significant effort is also needed to increase male partner involvement in adolescent women’s maternity care, delivery and follow-up. Again this requires a whole systems approach which first reduces the stigma of teenage pregnancy, increasing social supports around gender responsibility for offspring and engages men in ways which meet their needs for empowerment within the maternity care setting.

The review suggests that factors related to health providers such as negative attitudes and lack of training and sensitivity towards adolescent women’s needs influence the use of maternal health services by adolescent mothers in Sub-Saharan Africa. These findings are in line with previous studies which reported that adolescent mothers are sensitive to health providers’ attitudes and easily lose motivation to seek care [65, 66]. This review also indicates that adolescent mothers experience communication difficulties when interacting with care providers due to lack of privacy, confidentiality, and trust with health providers. As such, health providers need to give special attention to adolescent mothers as they are more likely to have others infections such as STIs and HIV and need more explanation of maternity care and motherhood compared to older mothers [41]. HCPs also need additional training to provide care that is sensitive and responsive to adolescent mothers’ needs [33].

Overall, research into the utilisation of maternal health service utilisation by adolescent women in Sub-Saharan Africa concentrates mainly on ANC with only a handful of studies addressing skilled birth delivery and PNC, which implies that additional research into adolescent women’s experiences of accessing skilled birth care and PNC is needed. This is critical as maternity service utilisation is the main window of opportunity for the provision of contraceptive services to prevent the recurrence of unplanned pregnancy among this vulnerable population. In addition, only one study has addressed the health provider views and experiences of providing maternity care to adolescent mothers which again suggests the need for more research into this area of maternity care. The majority of the studies also used quantitative designs involving a small number of adolescent women. As such the representativeness of these studies is questionable given the high rate of adolescent fertility in Sub-Saharan Africa. In response to this imperative, the research team is currently examining adolescent mother’s maternity service utilisation in Ethiopia using the large size, nationally representative demographic and health survey data.

### Limitations

This systematic review has a number of limitations that should be highlighted. The review may have publication bias as it considered only published articles and essential data from unpublished sources might have been missed. In addition, the studies included in this review lack power and representativeness due to small sample sizes. There are also more than 20 nations in Sub-Saharan Africa where French is an official language and the review may have missed significant data as it considered only articles written in English. This implies that generalisation of the findings to Sub-Saharan Africa may not be feasible.

## Conclusions

The review reveals that a significant number of adolescent women in Sub-Saharan Africa do not access and use maternity services during pregnancy. Several factors influence the maternity service utilisation patterns of adolescents which encompass individual, interpersonal, institutional, and systemic levels. This implies that interventions targeting the women, their partners, healthcare professionals and the system are necessary to improve adolescent mother’s engagement with maternity care in Sub-Saharan Africa. Notably, women need to be empowered through education and employment to improve their decision making autonomy about accessing care as having higher decision making freedom is associated with more ANC visits and skilled care during delivery. Access to care should also be improved especially for adolescents in rural areas, and increasing the number of providers would help to reduce waiting times and improve access to maternity care. Maternity services should also be enhanced through training of health providers to overcome prejudices that act as a barrier to utilisation. Finally, the provision of examination rooms and waiting areas that maintain privacy for adolescent mothers is required. These are just a few changes needed while systems and societies embark on the arduous journey of improving the health and wellbeing of teenage mothers with the aim of improving socioecological outcomes for whole communities and countries.

## Data Availability

The datasets used and/or analysed during the current study are available from the corresponding author on reasonable request.

## Abbreviations

ANC: Antenatal care
DHS: Demographic and Health Survey
EDHS: Ethiopian Demographic and Health Survey
HCP: Health Care Provider
ISPOR: International Society for Pharmacoeconomics and Outcomes Research
PMTCT: Prevention of mother to child transmission
PNC: Postnatal care
SRH: Sexual and Reproductive health
UN: United Nation
WHO: World Health Organization
